# K_2_[Fe^II^_3_(P_2_O_7_)_2_(H_2_O)_2_]

**DOI:** 10.1107/S1600536812021484

**Published:** 2012-05-19

**Authors:** Juan Yang, Xin Zhang, Biao Liu, Wei Sun, Ya-Xi Huang

**Affiliations:** a Department of Materials Science and Engineering, College of Materials, Xiamen University, Xiamen 361005, Fujian Province, People’s Republic of China

## Abstract

The title compound, dipotassium diaqua­bis­(diphosphato)triferrate(II), K_2_[Fe^II^_3_(P_2_O_7_)_2_(H_2_O)_2_], was synthesized under solvothermal conditions. The crystal structure is isotypic with its Co analogue. In the structure, there are two crystallographically distinct Fe positions; one lies on an inversion center, the other on a general position. The first Fe^2+^ cation adopts a regular octa­hedral coordination with six O atoms, whereas the other is coordinated by five O atoms and a water mol­ecule. The [FeO_6_] octa­hedron shares its *trans*-edges with an adjacent [FeO_5_(H_2_O)] octahedron; in turn, the [FeO_5_(H_2_O)] octa­hedron shares skew-edges with a neighbouring [FeO_6_] octa­hedron and an [FeO_5_(H_2_O)] octa­hedron, resulting in a zigzag octa­hedral chain running along [001]. The zigzag chains are linked to each other by the P_2_O_7_ diphosphate groups, leading to a corrugated iron diphosphate layer, [Fe_3_(P_2_O_7_)_2_(H_2_O)_2_]^2−^, parallel to (100). The inter­layer space is occupied by K^+^ cations, which adopt an eight-coordination to seven O atoms and one water mol­ecule from a neighbouring iron diphosphate layer. Thus, the K^+^ ions not only compensate the negative charge of the layer but also link the layers into a network structure.

## Related literature

For background to iron compounds, see: Mi *et al.* (2004 ▶); Huang *et al.* (2012 ▶). For related structures, see: Chippindale *et al.* (2003 ▶) for (NH_4_)_2_[Mn_3_(P_2_O_7_)_2_(H_2_O)_2_]; Lightfoot *et al.* (1990 ▶) for K_2_[Co_3_(P_2_O_7_)_2_(H_2_O)_2_]; Liu *et al.* (2012 ▶) for (NH_4_)_2_[Fe_3_^II^(P_2_O_7_(H_2_O)_2_)_2_]; Liu *et al.* (2004 ▶) for Na(NH_4_)[Ni_3_(P_2_O_7_)_2_(H_2_O)_2_]; Wei *et al.* (2010 ▶) for (NH_4_)_2_[Ni_3_(P_2_O_7_)_2_(H_2_O)_2_]. For bond-valence calculations, see: Brown (2002 ▶).

## Experimental

### 

#### Crystal data

K_2_[Fe_3_(P_2_O_7_)_2_(H_2_O)_2_]
*M* *_r_* = 629.66
Monoclinic, 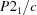

*a* = 9.1517 (16) Å
*b* = 8.1737 (15) Å
*c* = 9.3147 (17) Å
β = 98.860 (3)°
*V* = 688.5 (2) Å^3^
*Z* = 2
Mo *K*α radiation
μ = 4.28 mm^−1^
*T* = 173 K
0.09 × 0.09 × 0.08 mm

#### Data collection

Bruker SMART CCD area-detector diffractometer
Absorption correction: multi-scan (*SMART*; Bruker, 2001 ▶) *T*_min_ = 0.687, *T*_max_ = 0.710
4006 measured reflections
1596 independent reflections
1460 reflections with *I* > 2σ(*I*)
*R*_int_ = 0.027

#### Refinement

*R*[*F* ^2^ > 2σ(*F* ^2^)] = 0.032
*wR*(*F* ^2^) = 0.074
*S* = 1.07
1596 reflections
123 parameters
All H-atom parameters refined
Δρ_max_ = 0.57 e Å^−3^
Δρ_min_ = −0.66 e Å^−3^

### 

Data collection: *SMART* (Bruker, 2001 ▶); cell refinement: *SAINT* (Bruker, 2001 ▶); data reduction: *SAINT*; program(s) used to solve structure: *SHELXS97* (Sheldrick, 2008 ▶); program(s) used to refine structure: *SHELXL97* (Sheldrick, 2008 ▶); molecular graphics: *DIAMOND* (Brandenburg, 2005 ▶) and *ATOMS* (Dowty, 2004 ▶); software used to prepare material for publication: *SHELXL97* and *WinGX* (Farrugia, 1999 ▶).

## Supplementary Material

Crystal structure: contains datablock(s) I, global. DOI: 10.1107/S1600536812021484/br2202sup1.cif

Structure factors: contains datablock(s) I. DOI: 10.1107/S1600536812021484/br2202Isup2.hkl

Additional supplementary materials:  crystallographic information; 3D view; checkCIF report

## Figures and Tables

**Table 1:** Hydrogen-bond geometry (Å, °)

*D*—H⋯*A*	*D*—H	H⋯*A*	*D*⋯*A*	*D*—H⋯*A*
O8—H1⋯O3^i^	0.82 (5)	1.90 (5)	2.716 (4)	171 (5)
O8—H2⋯O7^ii^	0.76 (4)	1.95 (4)	2.696 (4)	164 (4)

Symmetry codes: (i) 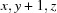; (ii) 
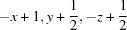
.

**Fig. 1.**
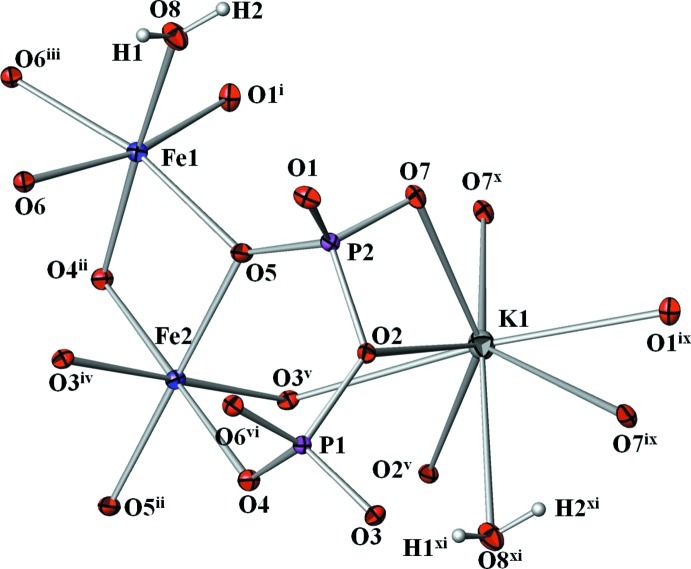
Structural unit of K2[FeII3(P2O7)2(H2O)2], showing the coordination environments of Fe, K, and P atoms. Thermal ellipsoids were drawn at the 50% probability level. Symmetry codes: (i) x, –y+3/2, z+1/2; (ii) –x+2, –y+1, –z+1; (iii) –x+2,–y+2, –z+1; (iv) –x+2, y+1/2, –z+1/2; (v) x, –y+1/2, z+1/2; (vi) –x+2, y–1/2, –z+1/2; (vii) x, –y+1/2, z–1/2; (viii) –x+1, y+1/2, –z+1/2; (ix) x+1, y–1/2, –z+1/2; (x) –x+1, –y+1, –z+1; (xi) x, y–1, z; (xii) x, –y+3/2, z–1/2; (xiii) x, y+1, z.

**Fig. 2.**
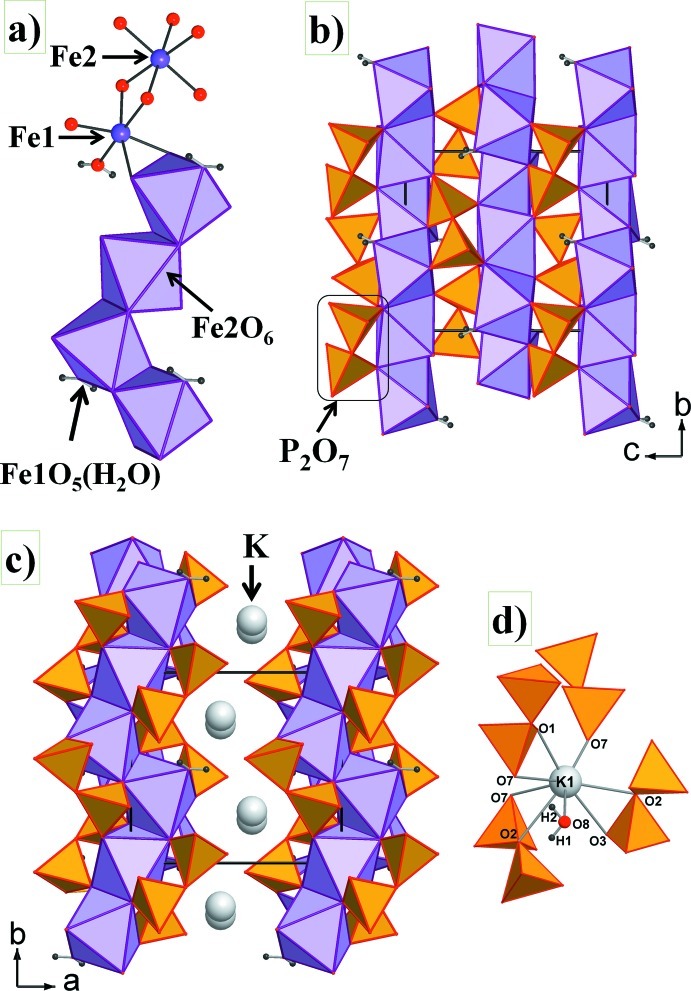
Polyhedral presentation of the crystal structure of K2[FeII3(P2O7)2(H2O)2], (a) zigzag edge-sharing iron octahedral chain is built from FeO6 and FeO5(H2O) octahedra by sharing their trans- or skew-edges running along [010]; (b) P2O7 groups act as a bidentate ligand to link FeO6-based chains along [001] to form a corrugated iron diphosphate layer [Fe3(P2O7)2(H2O)2]2–; (c) the iron diphosphate layers stack along [100] in AAA fashion with the potassium atoms locating at the interlayer space; (d) the K atom is 8-coordinated to seven oxygen atoms and a H2O molecule coming from adjacent iron diphosphate layers to form an irregular polyhedron. Purple octahedron: FeO6, orange tetrahedron: PO4, light grey sphere: K atom, dark grey sphere: H atom.

## supplementary crystallographic information

### Comment

A class of layered transition metal diphosphates with the general formula of *A*_2_*M*_3_(P_2_O_7_)_2_(H_2_O)_2_ (*A* = K or NH_4_, *M* = transition metal (II)) has recently attracted much attention. Since the first publication of K_2_Co_3_(P_2_O_7_)_2_(H_2_O)_2_ (Lightfoot *et al.*, 1990) in 1990, a few examples of ammonium substituted compounds, (NH_4_)_2_*M*_3_(P_2_O_7_)_2_(H_2_O)_2_ (*M* = Mn^2+^, Ni^2+^) (Chippindale *et al.*, 2003; Wei *et al.*, 2010), have been synthesized by using either solvothermal or ionothermal method. Their magnetic and ion exchange behaviors have also been discussed. Besides, a compound with mixed cations, Na(NH_4_)[Ni_3_(P_2_O_7_)_2_(H_2_O)_2_], has also been reported (Liu *et al.*, 2004). Although compounds incorporating with Mn^2+^, Co^2+^, and Ni^2+^ have been known, as a very close relationship to these elements, Fe^2+^ was not yet found in this class of compounds until very recently an ammonium iron diphosphate, (NH_4_)_2_[Fe_3_^II^(P_2_O_7_)_2_(H_2_O)_2_], was reported by our group which was synthesized by using iron powder as iron source and applying the weak reductive agent pyridine as solvent (Liu *et al.*, 2012). During our systematically investigation on iron compounds (Mi *et al.*, 2004; Huang *et al.*, 2012), we obtained a new member in this class of compounds with the formula of K_2_[Fe^II^_3_(P_2_O_7_)_2_(H_2_O)_2_].

In the crystal structure of the title compound, there are two crystallographically distinct iron sites, one potassium, and two independent phosphorus (shown in Figure 1). Fe(2) lies on an inversion center (2*a*, -1) while Fe(1) on a general position (4*e*, 1). Fe(2) adopts a regular octahedral coordination with six oxygen atoms, whereas Fe(1) coordinates to five oxygen atoms and a water molecule in the form of Fe(1)O_5_(OH_2_). Each Fe(2)O_6_ octahedron shares trans-edges with adjacent Fe(1)O_5_(OH_2_) octahedra, and in turn, Fe(1)O_5_(OH_2_) octahedron shares skew-edges with the neighboring Fe(2)O_6_ and Fe(1)O_5_(OH_2_) octahedra, which finally leads to the formation of FeO_6_-based zigzag chains parallel to [010] (see Figure 2a). Two independent phosphorus atoms adopt a four-fold coordination with one long P–O bond and three general P–O bonds which are often found in diphosphates. Every two PO_4_ tetrahedra share the common O-vertex (O2) to constitute a P_2_O_7_ group which acts as a bidentate ligand to link FeO_6_-based chains along [001], resulting in a undulating iron diphosphate layer, [Fe_3_(P_2_O_7_)_2_(H_2_O)_2_]^2–^, parallel to the (100) (see Figure 2b). The layers stack along the *a*-axis direction in AAA fashion with the potassium atoms locating at the interlayer space (see Figure 2c). The potassium ion is 8-coordinated with seven oxygens and a water molecule from the neighbouring layers with one long bond and seven relatively short bonds (see Figure 2d). Bond-valence sum calculations (Brown, 2002) suggests both two iron atoms are in the 2+ valence state (Cald.: 2.008 v.u. for Fe(1) and 2.112 v.u. for Fe(2) and the potassium should be +1 oxidation (Cald.: 1.054 v.u. for K1). Comparing the crystal structure of NH_4_- and K-compounds, both of them possess the same conformation of iron diphosphate layer, however, the interlayer distance of K-compound is relatively smaller than that of NH_4_-compound identified by the *a*-value (9.1517 (16) Å for KFe-compound and 9.4131 (17) Å for NH_4_Fe-compound) which is consistent with the ion radius of K^+^ and NH_4_^+^.

### Experimental

The title compound has been synthesized under solvothermal condition. In a typical synthesis, 1 mmol Fe powder and 2.5 mmol KH_2_PO_4_ were added into the mixed solvent of 2 mL pyridine and 2 mL 1,2-propanediol. Then, 2 mL H_3_PO_4_ (85%) was dropped into the above solution to adjust pH value to 6-7. After stirred for 5 minutes, the mixture was transferred into a 15 mL Teflon-lined stainless steel autoclave which was heated at 436 K for 7 days and then was cooled down to room temperature. The final product, colourless block-like crystals, was washed with distilled water and filtrated by vacuum. The powder X-ray diffraction of the sample showed that it is isotypic to our recently reported compound [NH_4_]_2_[Fe^II^_3_(H_2_O)_2_(P_2_O_7_)_2_].

### Refinement

The hydrogen atoms bonded to water (O8) were located from the difference Fourier maps and refined without applying any constraints on the distance of O–H and the displacement parameter of H atoms.

### Crystal data

**Table:**

K_2_[Fe_3_(P_2_O_7_)_2_(H_2_O)_2_]	*F*(000) = 616
*M**_r_* = 629.66	*D*_x_ = 3.037 Mg m^−^^3^
Monoclinic, *P*2_1_/*c*	Mo *K*α radiation, λ = 0.71073 Å
Hall symbol: -P 2ybc	Cell parameters from 4006 reflections
*a* = 9.1517 (16) Å	θ = 2.3–28.3°
*b* = 8.1737 (15) Å	µ = 4.28 mm^−^^1^
*c* = 9.3147 (17) Å	*T* = 173 K
β = 98.860 (3)°	Block, colourless
*V* = 688.5 (2) Å^3^	0.09 × 0.09 × 0.08 mm
*Z* = 2	

### Data collection

**Table:**

Bruker SMART CCD area-detector diffractometer	1596 independent reflections
Radiation source: fine-focus sealed tube	1460 reflections with *I* > 2σ(*I*)
Graphite monochromator	*R*_int_ = 0.027
1265 images,φ=0, 90, 180 degree, and Δω=0.3 degree, χ= 54.74 degree scans	θ_max_ = 28.3°, θ_min_ = 2.3°
Absorption correction: multi-scan (*SMART*; Bruker, 2001)	*h* = −11→12
*T*_min_ = 0.687, *T*_max_ = 0.710	*k* = −10→10
4006 measured reflections	*l* = −12→8

### Refinement

**Table:**

Refinement on *F*^2^	Primary atom site location: structure-invariant direct methods
Least-squares matrix: full	Secondary atom site location: difference Fourier map
*R*[*F*^2^ > 2σ(*F*^2^)] = 0.032	Hydrogen site location: difference Fourier map
*wR*(*F*^2^) = 0.074	All H-atom parameters refined
*S* = 1.07	*w* = 1/[σ^2^(*F*_o_^2^) + (0.0303*P*)^2^ + 1.280*P*] where *P* = (*F*_o_^2^ + 2*F*_c_^2^)/3
1596 reflections	(Δ/σ)_max_ < 0.001
123 parameters	Δρ_max_ = 0.57 e Å^−^^3^
0 restraints	Δρ_min_ = −0.66 e Å^−^^3^

### Special details

**Table:**

Geometry. All esds (except the esd in the dihedral angle between two l.s. planes) are estimated using the full covariance matrix. The cell esds are taken into account individually in the estimation of esds in distances, angles and torsion angles; correlations between esds in cell parameters are only used when they are defined by crystal symmetry. An approximate (isotropic) treatment of cell esds is used for estimating esds involving l.s. planes.
Refinement. Refinement of F^2^ against ALL reflections. The weighted R-factor wR and goodness of fit S are based on F^2^, conventional R-factors R are based on F, with F set to zero for negative F^2^. The threshold expression of F^2^ > 2sigma(F^2^) is used only for calculating R-factors(gt) etc. and is not relevant to the choice of reflections for refinement. R-factors based on F^2^ are statistically about twice as large as those based on F, and R- factors based on ALL data will be even larger.

### Fractional atomic coordinates and isotropic or equivalent isotropic displacement parameters (Å^2^)

**Table:**

	*x*	*y*	*z*	*U*_iso_*/*U*_eq_	
Fe1	0.86324 (5)	0.85888 (6)	0.50352 (5)	0.00807 (13)	
Fe2	1.0000	0.5000	0.5000	0.00754 (16)	
P1	0.89966 (9)	0.30110 (10)	0.20949 (9)	0.00652 (18)	
P2	0.69498 (9)	0.56235 (10)	0.26759 (9)	0.00744 (18)	
K1	0.57440 (8)	0.27162 (10)	0.48169 (9)	0.01682 (19)	
O1	0.6935 (3)	0.6515 (3)	0.1248 (3)	0.0111 (5)	
O2	0.7397 (2)	0.3719 (3)	0.2360 (2)	0.0090 (5)	
O3	0.8654 (2)	0.1362 (3)	0.1376 (2)	0.0084 (5)	
O4	0.9925 (2)	0.2944 (3)	0.3595 (2)	0.0094 (5)	
O5	0.8140 (2)	0.6235 (3)	0.3887 (3)	0.0103 (5)	
O6	1.0389 (2)	0.9230 (3)	0.3891 (2)	0.0089 (5)	
O7	0.5448 (2)	0.5477 (3)	0.3129 (3)	0.0120 (5)	
O8	0.7250 (3)	0.9942 (3)	0.3420 (3)	0.0146 (5)	
H1	0.770 (5)	1.027 (6)	0.278 (5)	0.023 (13)*	
H2	0.645 (5)	0.991 (5)	0.302 (5)	0.013 (11)*	

### Atomic displacement parameters (Å^2^)

**Table:**

	*U*^11^	*U*^22^	*U*^33^	*U*^12^	*U*^13^	*U*^23^
Fe1	0.0087 (2)	0.0085 (2)	0.0070 (2)	0.00054 (17)	0.00131 (17)	0.00043 (17)
Fe2	0.0088 (3)	0.0075 (3)	0.0061 (3)	0.0003 (2)	0.0004 (2)	0.0000 (2)
P1	0.0070 (4)	0.0068 (4)	0.0058 (4)	0.0001 (3)	0.0008 (3)	−0.0002 (3)
P2	0.0072 (4)	0.0081 (4)	0.0069 (4)	0.0007 (3)	0.0007 (3)	−0.0007 (3)
K1	0.0152 (4)	0.0183 (4)	0.0164 (4)	−0.0009 (3)	0.0007 (3)	0.0013 (3)
O1	0.0113 (11)	0.0142 (12)	0.0083 (11)	0.0030 (9)	0.0029 (9)	0.0019 (9)
O2	0.0072 (11)	0.0086 (11)	0.0113 (12)	−0.0005 (8)	0.0015 (9)	−0.0025 (9)
O3	0.0105 (11)	0.0075 (11)	0.0071 (11)	−0.0004 (8)	0.0014 (9)	−0.0016 (9)
O4	0.0103 (11)	0.0096 (11)	0.0079 (11)	0.0005 (9)	0.0002 (9)	−0.0006 (9)
O5	0.0096 (11)	0.0089 (11)	0.0114 (12)	0.0017 (9)	−0.0014 (9)	−0.0020 (9)
O6	0.0091 (11)	0.0094 (12)	0.0082 (11)	−0.0001 (9)	0.0021 (9)	−0.0014 (9)
O7	0.0072 (11)	0.0134 (12)	0.0158 (13)	−0.0004 (9)	0.0028 (9)	−0.0008 (10)
O8	0.0097 (13)	0.0200 (14)	0.0140 (14)	0.0006 (10)	0.0009 (11)	0.0061 (10)

### Geometric parameters (Å, º)

**Table:**

Fe1—O1^i^	2.058 (2)	P1—O2	1.628 (2)
Fe1—O4^ii^	2.103 (2)	P2—O7	1.503 (2)
Fe1—O8	2.121 (3)	P2—O1	1.515 (2)
Fe1—O6	2.127 (2)	P2—O5	1.527 (2)
Fe1—O6^iii^	2.169 (2)	P2—O2	1.648 (2)
Fe1—O5	2.214 (2)	K1—O1^vii^	2.685 (2)
Fe2—O5^ii^	2.109 (2)	K1—O7	2.740 (3)
Fe2—O5	2.109 (2)	K1—O7^viii^	2.771 (3)
Fe2—O4^ii^	2.124 (2)	K1—O2^iv^	2.859 (2)
Fe2—O4	2.124 (2)	K1—O3^iv^	2.929 (2)
Fe2—O3^iv^	2.210 (2)	K1—O2	3.043 (2)
Fe2—O3^v^	2.210 (2)	K1—O8^ix^	3.047 (3)
P1—O3	1.516 (2)	K1—O7^vii^	3.339 (3)
P1—O6^vi^	1.520 (2)	O8—H1	0.82 (5)
P1—O4	1.521 (2)	O8—H2	0.76 (4)
			
O1^i^—Fe1—O4^ii^	95.66 (9)	O1^vii^—K1—O7	94.87 (7)
O1^i^—Fe1—O8	89.59 (10)	O1^vii^—K1—O7^viii^	90.91 (7)
O4^ii^—Fe1—O8	172.30 (10)	O7—K1—O7^viii^	86.72 (8)
O1^i^—Fe1—O6	167.91 (9)	O1^vii^—K1—O2^iv^	119.53 (7)
O4^ii^—Fe1—O6	89.92 (9)	O7—K1—O2^iv^	143.75 (7)
O8—Fe1—O6	86.00 (10)	O7^viii^—K1—O2^iv^	81.99 (7)
O1^i^—Fe1—O6^iii^	94.22 (9)	O1^vii^—K1—O3^iv^	170.43 (7)
O4^ii^—Fe1—O6^iii^	91.94 (9)	O7—K1—O3^iv^	94.29 (7)
O8—Fe1—O6^iii^	93.29 (10)	O7^viii^—K1—O3^iv^	86.85 (7)
O6—Fe1—O6^iii^	74.83 (10)	O2^iv^—K1—O3^iv^	50.94 (6)
O1^i^—Fe1—O5	96.59 (9)	O1^vii^—K1—O2	110.62 (7)
O4^ii^—Fe1—O5	80.63 (9)	O7—K1—O2	50.46 (7)
O8—Fe1—O5	93.20 (10)	O7^viii^—K1—O2	132.06 (7)
O6—Fe1—O5	94.90 (9)	O2^iv^—K1—O2	118.20 (8)
O6^iii^—Fe1—O5	167.42 (9)	O3^iv^—K1—O2	77.53 (6)
O5^ii^—Fe2—O5	180.0	O1^vii^—K1—O8^ix^	91.01 (8)
O5^ii^—Fe2—O4^ii^	97.39 (9)	O7—K1—O8^ix^	112.28 (8)
O5—Fe2—O4^ii^	82.61 (9)	O7^viii^—K1—O8^ix^	160.66 (8)
O5^ii^—Fe2—O4	82.61 (9)	O2^iv^—K1—O8^ix^	80.33 (7)
O5—Fe2—O4	97.39 (9)	O3^iv^—K1—O8^ix^	88.08 (7)
O4^ii^—Fe2—O4	180.0	O2—K1—O8^ix^	64.50 (7)
O5^ii^—Fe2—O3^iv^	87.34 (9)	O1^vii^—K1—O7^vii^	48.08 (6)
O5—Fe2—O3^iv^	92.66 (9)	O7—K1—O7^vii^	89.37 (3)
O4^ii^—Fe2—O3^iv^	90.54 (9)	O7^viii^—K1—O7^vii^	138.28 (9)
O4—Fe2—O3^iv^	89.46 (9)	O2^iv^—K1—O7^vii^	121.35 (7)
O5^ii^—Fe2—O3^v^	92.66 (9)	O3^iv^—K1—O7^vii^	134.87 (7)
O5—Fe2—O3^v^	87.34 (9)	O2—K1—O7^vii^	70.56 (6)
O4^ii^—Fe2—O3^v^	89.46 (9)	O8^ix^—K1—O7^vii^	49.70 (7)
O4—Fe2—O3^v^	90.54 (9)	P2—O1—Fe1^x^	123.83 (14)
O3^iv^—Fe2—O3^v^	180.0	P1—O2—P2	128.09 (15)
O3—P1—O6^vi^	112.74 (13)	P1—O3—Fe2^vi^	127.45 (13)
O3—P1—O4	114.99 (13)	P1—O4—Fe1^ii^	142.06 (15)
O6^vi^—P1—O4	111.86 (13)	P1—O4—Fe2	119.89 (13)
O3—P1—O2	104.68 (12)	Fe1^ii^—O4—Fe2	98.04 (9)
O6^vi^—P1—O2	106.47 (13)	P2—O5—Fe2	129.55 (14)
O4—P1—O2	105.14 (13)	P2—O5—Fe1	135.27 (14)
O7—P2—O1	113.58 (13)	Fe2—O5—Fe1	95.14 (9)
O7—P2—O5	113.47 (14)	P1^v^—O6—Fe1	121.29 (13)
O1—P2—O5	113.57 (14)	P1^v^—O6—Fe1^iii^	130.73 (13)
O7—P2—O2	103.75 (13)	Fe1—O6—Fe1^iii^	105.17 (10)
O1—P2—O2	105.48 (13)	H1—O8—H2	102 (4)
O5—P2—O2	105.80 (13)		

Symmetry codes: (i) *x*, −*y*+3/2, *z*+1/2; (ii) −*x*+2, −*y*+1, −*z*+1; (iii) −*x*+2, −*y*+2, −*z*+1; (iv) *x*, −*y*+1/2, *z*+1/2; (v) −*x*+2, *y*+1/2, −*z*+1/2; (vi) −*x*+2, *y*−1/2, −*z*+1/2; (vii) −*x*+1, *y*−1/2, −*z*+1/2; (viii) −*x*+1, −*y*+1, −*z*+1; (ix) *x*, *y*−1, *z*; (x) *x*, −*y*+3/2, *z*−1/2.

### Hydrogen-bond geometry (Å, º)

**Table:**

*D*—H···*A*	*D*—H	H···*A*	*D*···*A*	*D*—H···*A*
O8—H1···O3^xi^	0.82 (5)	1.90 (5)	2.716 (4)	171 (5)
O8—H2···O7^xii^	0.76 (4)	1.95 (4)	2.696 (4)	164 (4)

Symmetry codes: (xi) *x*, *y*+1, *z*; (xii) −*x*+1, *y*+1/2, −*z*+1/2.
